# Diagnostic delay and predictors of true stroke among patients with suspected in-hospital stroke: a single-center retrospective study

**DOI:** 10.3389/fneur.2026.1979991

**Published:** 2026-10-22

**Authors:** Koji Tanaka, Ko Nakamura, Yuina Mori, Junpei Koge, Tetsuya Hashimoto, Eiji Fujiwara, Kiyonori Kuwahara, Mitsunaga Iwata, Tomoyuki Nakamura, Yuichi Hirose, Shoji Matsumoto

**Affiliations:** 1 Department of Comprehensive Strokology, Fujita Health University, Toyoake, Japan; 2 School of Medicine, Fujita Health University, Toyoake, Japan; 3 Department of Neurosurgery, Fujita Health University, Toyoake, Japan; 4 Department of Emergency and General Internal Medicine, Fujita Health University, Toyoake, Japan; 5 Department of Anesthesiology and Critical Care Medicine, Fujita Health University, Toyoake, Japan

**Keywords:** delay, imaging, in-hospital stroke, reperfusion therapy, stroke mimics, time metrics

## Abstract

**Background:**

Timely diagnosis is essential for acute treatment of in-hospital stroke. We aimed to investigate factors associated with diagnostic delay and clinical predictors of true stroke among patients with suspected in-hospital stroke.

**Methods:**

In this single-center retrospective study, we screened head computed tomography and magnetic resonance imaging orders between Jan 2023 and Jan 2026 for patients hospitalized outside the Departments of Neurology and Neurosurgery who developed new neurological symptoms within 24 h of symptom onset or last known well and were clinically suspected of having in-hospital stroke. Multivariable analyses were performed to identify factors associated with diagnostic delay and true stroke.

**Results:**

A total of 183 patients (110 men, median age 77 years) were included. Median time from last known well to imaging-based final diagnosis was 292 min. Overall, 77 patients (42.1%) had true stroke, including 70 ischemic strokes, 5 intracerebral hemorrhages, and 2 subarachnoid hemorrhages. Wake-up symptom onset [Exp (β) 3.34, 95% confidence interval (CI) 2.61–4.28] and initial medical emergency team activation [Exp (β) 0.71, 95% CI 0.56–0.91] were associated with diagnostic delay. Persistent neurological symptoms [odds ratio (OR) 1.44, 95% CI 1.24–1.68] and FAST-positive symptoms (OR 1.28, 95% CI 1.12–1.46) were associated with true stroke. Among the 70 patients with ischemic stroke, 17 (24.3%) underwent reperfusion therapy.

**Conclusion:**

Wake-up symptom onset and medical emergency team activation were associated with diagnostic delay, whereas persistent and FAST-positive symptoms were associated with true stroke. These findings may facilitate earlier recognition and improve triaging patients with suspected in-hospital stroke.

## Introduction

In-hospital stroke is associated with severe neurological deficits, high mortality, and poor functional outcomes (1, 2). Previous studies showed that in-hospital stroke is associated with longer times to neuroimaging and acute treatment, lower rates of reperfusion therapy, and poorer outcomes compared with out-of-hospital stroke (3–5). In addition, hospitalized patients frequently have multiple comorbidities, recent surgery status, altered consciousness, or medication effects, making recognition of acute stroke symptoms challenging (6, 7).

Several workflow-related factors may contribute to diagnostic delay in in-hospital stroke. Patients may develop symptoms during while asleep when neurological monitoring is less frequent, and potential barriers to urgent neurological consultation or emergent neuroimaging may further delay diagnosis (8–10). However, previous studies have mainly focused on confirmed cases of in-hospital stroke, and little is known about the broader population of patients with suspected in-hospital stroke encountered in real-world clinical practice. Moreover, hospitalized patients may frequently develop stroke-like neurological symptoms due to underlying medical conditions, potentially increasing the proportion of stroke mimics in this setting (6, 7). Identifying clinical characteristics and presenting neurological symptoms associated with true stroke may facilitate earlier recognition and appropriate triage, particularly in non-stroke-specialized departments.

Therefore, we aimed to investigate (1) factors associated with diagnostic delay among patients hospitalized outside the Departments of Neurology and Neurosurgery who developed new neurological symptoms suspicious for stroke, and (2) clinical characteristics and presenting neurological symptoms associated with true stroke among patients with suspected in-hospital stroke.

## Materials and methods

### Study design

In this single-center retrospective study, we extracted all head computed tomography (CT) and magnetic resonance imaging (MRI) orders placed between Jan 2023 and Jan 2026 for patients hospitalized outside the Neurology and Neurosurgery departments at Fujita Health University Hospital. Our hospital is a 1,376-bed tertiary academic center serving the eastern part of the Nagoya metropolitan area. Imaging orders were screened by KN and YM, medical students, to identify examinations requested for the diagnosis or exclusion of suspected acute in-hospital stroke. Medical records of patients identified through this screening process were subsequently reviewed by KT, an experienced stroke neurologist, and patients were included if they had newly recognized neurological symptoms within 24 h of symptom onset or last known well. Neuroimaging was ordered at the discretion of the attending physicians in each department. The medical emergency team (MET), consisting of physicians and nurses from the intensive care unit, was available 24/7 to assist in the management of inpatient emergencies. No standardized institutional imaging protocol for evaluating suspected in-hospital stroke was in place during the study period. Non-contrast CT (NCCT) was routinely performed as the initial imaging modality because of its availability. Additional imaging, including CT angiography (CTA) or MRI, was performed when clinically indicated. Neurological consultation was generally requested after imaging confirmed stroke, and subsequent treatment decisions were made by attending neurologists or neurosurgeons. Acute reperfusion therapy, including intravenous thrombolysis (IVT) and/or endovascular therapy (EVT), was performed in accordance with the Japanese guidelines (11). We used the STROBE reporting guideline (12) to draft this manuscript, and the STROBE reporting checklist (13) when editing.

### Study participants

The following clinical information was collected from medical records: age, sex, vascular risk factors (hypertension, diabetes mellitus, dyslipidemia, atrial fibrillation, and prior stroke), active malignancy, maintenance hemodialysis, and prior use of antiplatelet or anticoagulant agents. Workflow-related variables included timing of symptom detection [daytime working hours (8:00 a.m.–5:00 p.m.) or nighttime hours (5:00 p.m.–8:00 a.m.)], first person detecting symptoms (nurses, physicians, or other allied health professionals), initial activation of MET, and admitting department. Admitting departments were categorized as internal medicine, surgical departments, or others (Supplementary Table 1). Time metrics were calculated from time stamps of the medical record. Wake-up symptom onset was defined as neurological symptoms first recognized after awakening or following a prolonged interval without neurological assessment. When symptom onset was unknown, the time of last known well was defined as the last documented time at which the patient was known to be neurologically unchanged before symptom detection. When the exact time of symptom onset was unavailable, it was defined as the first documented time of newly recognized neurological symptoms in the medical record. Time of initial neuroimaging was obtained from the timestamp of the first CT or MRI examination. Time of final diagnosis was defined as the time at which neuroimaging findings became sufficient to establish the diagnosis and guide treatment decisions. Glasgow Coma Scale score at symptom onset was recorded when available. Neurological symptoms were categorized as decreased consciousness, any motor symptoms, any speech symptoms, any sensory symptoms, and/or others. FAST-positive symptoms were defined as the presence of any motor and/or speech symptoms. Transient symptoms were defined as neurological symptoms that had completely resolved before the completion of neuroimaging required to establish the final diagnosis; all other symptoms were classified as persistent. All neuroimaging findings were reviewed, and final diagnoses were categorized as ischemic stroke, intracerebral hemorrhage, subarachnoid hemorrhage, or non-stroke. National Institutes of Health Stroke Scale (NIHSS) scores were collected for patients with ischemic stroke when available.

### Outcomes

The primary metric of interest was the interval from last known well/symptom onset to imaging-based final diagnosis. Secondary metrics of interest were intervals from last known well/symptom onset to symptom detection and from symptom detection to initial neuroimaging. The diagnostic outcome was categorized as true stroke (ischemic stroke, intracerebral hemorrhage, or subarachnoid hemorrhage) or non-stroke.

### Statistical analysis

Statistical analyses were performed using Stata version 18.0 (StataCorp, College Station, TX, United States). Continuous variables are presented as medians with interquartile ranges (IQRs), and categorical variables as counts with percentages. Group comparisons were performed using Pearson’s chi-square test or Fisher’s exact test for categorical variables and the Wilcoxon rank-sum test for continuous variables. Factors associated with diagnostic delay were investigated using the interval from last known well to imaging-based final diagnosis as the dependent variable. The diagnostic time was log-transformed before regression analyses. Regression coefficients were exponentiated and reported as ratios of diagnostic time. Multivariable linear regression was performed for diagnostic time metrics. Candidate variables were prespecified based on clinical relevance. Given the limited sample size relative to the number of candidate variables, stepwise selection was used to reduce model overfitting. Age and sex were included in all models regardless of statistical significance. Candidate variables considered clinically relevant to diagnostic delay (wake-up symptom onset, first person detecting symptoms, initial MET activation, timing of symptom detection, admitting department, and requirement for additional imaging) were entered into a stepwise selection procedure (entry *p* < 0.20, removal *p* > 0.10). Adjusted estimated diagnostic times were calculated for patients with and without wake-up symptom onset from multivariable models using marginal predictions. Additional analyses were performed for the intervals from last known well/symptom onset to symptom detection and from symptom detection to initial neuroimaging using the same covariates. A similar multivariable analysis was performed for the time from symptom detection to imaging-based final diagnosis. Multivariable logistic regression was performed to identify factors associated with true stroke. Age and sex were forced into all models, whereas candidate variables (hypertension, diabetes mellitus, dyslipidemia, atrial fibrillation, history of stroke, active malignancy, maintenance hemodialysis, prior use of antiplatelet or anticoagulant agents, persistent symptoms, and FAST-positive symptoms) were selected using same stepwise selection procedure (entry *p* < 0.20, removal *p* > 0.10). Additional analyses were performed to investigate associations between each presenting neurological symptom (loss of consciousness, motor symptoms, speech symptoms, sensory symptoms, and/or other symptoms) and true stroke using the same covariates as in the primary model. Complete case analysis was performed. All statistical tests were two-sided, and *p* values <0.05 were considered statistically significant.

## Results

A total of 183 patients (110 men, median age 77 years) were included (Supplementary Figure 1). Baseline characteristics and time metrics according to wake-up symptom onset and true stroke status are summarized in Tables 1, 2, respectively. Initial neuroimaging consisted of NCCT in 153 patients, CTA in 4, and MRI in 26. Among the 154 patients who underwent NCCT first, additional imaging was required in 120 patients, including MRI in 119 and CTA in 1. Overall, MRI was ultimately required in 145 of 183 patients (79.2%) to establish the diagnosis. Median intervals from last known well/symptom onset to symptom detection, symptom detection to initial neuroimaging, and last known well/symptom onset to imaging-based final diagnosis were 120 (IQR 41–507), 69 (39–151), and 295 (162–796) minutes, respectively. In multivariable analysis, wake-up symptom onset [Exp (β) 3.34, 95% confidence interval (CI) 2.61–4.28] was associated with longer diagnostic time, whereas initial MET activation [Exp (β) 0.71, 95% CI 0.56–0.91] was associated with shorter diagnostic time (Table 3). Adjusted estimated time metrics according to wake-up symptom onset are shown in Supplementary Figure 2. The results of the multivariable analysis of the time from symptom detection to imaging-based final diagnosis are shown in Supplementary Table S2.

**Table 1:** Time metrics and workflow related factors in patients with and without wake-up symptom onset.

Variables	Wake-up symptom onset		*p* value
	Yes (*n* = 53)	No (*n* = 130)	
Age in years	75 (62–81)	78 (69–83)	0.16
Sex, men	28 (52.8%)	82 (63.1%)	0.20
Admitting department			0.66
Internal medicine	35 (66.0%)	77 (59.2%)	
Surgery	13 (24.5%)	36 (27.7%)	
Others	5 (9.4%)	17 (13.1%)	
Timing of symptom detection, daytime working hours	37 (69.8%)	95 (73.1%)	0.66
First person detecting symptoms			0.05
Nurses	45 (84.9%)	105 (80.8%)	
Physicians	8 (15.1%)	13 (10.0%)	
Other allied health professionals	0 (0.0%)	12 (9.2%)	
Initial MET activation	18 (34.0%)	44 (33.8%)	0.99
Additional neuroimaging required	33 (62.3%)	87 (67.0%)	0.69
Time from last known well/symptom onset to detection	615 (505–693)	73 (20–135)	<0.01
Symptom detection to initial neuroimaging	86 (52–195)	55 (35–124)	0.01
Symptom detection to imaging-based final diagnosis	158 (91–290)	127 (77–207)	0.04
Time from last known well/symptom onset to imaging-based final diagnosis	833 (638–997)	209 (134–342)	<0.01

Data were presented by *n* (%) or median (interquartile range). MET, medical emergency team.

**Table 2:** Patient characteristics in patients with and without final diagnosis of true stroke.

Variables	Final diagnosis of stroke		*p* value
	Yes (*n* = 77)	No (*n* = 106)	
Age in years	76 (65–82)	78 (70–83)	0.28
Sex, men	48 (62.3%)	62 (58.5%)	0.60
Hypertension	36 (46.7%)	49 (46.2%)	0.94
Diabetes mellitus	26 (33.8%)	37 (34.9%)	0.87
Dyslipidemia	15 (19.5%)	29 (27.4%)	0.22
Atrial fibrillation	23 (29.9%)	14 (13.2%)	<0.01
History of stroke	15 (19.5%)	36 (34.0%)	0.03
Active malignancy	10 (13.0%)	24 (22.6%)	0.10
Maintenance hemodialysis	8 (10.4%)	23 (21.7%)	0.04
Prior antiplatelet therapy	27 (35.1%)	40 (37.7%)	0.71
Prior anticoagulant therapy	13 (16.9%)	16 (15.1%)	0.74
Glasgow Coma Scale score at onset*	13 (9–15)	13 (10–15)	0.68
Presenting neurological symptoms			
FAST-positive symptoms	55 (71.4%)	42 (39.6%)	<0.01
Loss of consciousness	37 (48.1%)	57 (53.8%)	0.45
Motor symptoms	41 (53.2%)	30 (28.3%)	<0.01
Speech symptoms	25 (32.5%)	18 (17.0%)	0.02
Sensory symptoms	4 (5.2%)	12 (11.3%)	0.15
Other symptoms	8 (10.4%)	13 (12.3%)	0.69
Transient symptoms	5 (6.5%)	40 (37.7%)	<0.01
Time from last known well/symptom onset to detection	134 (62–524)	111 (30–482)	0.20
Symptom detection to initial neuroimaging	62 (38–131)	75 (40–163)	0.40
Symptom detection to imaging-based final diagnosis	106 (70–217)	156 (81–258)	0.07
Time from last known well/symptom onset to imaging-based final diagnosis	325 (161–772)	277 (168–850)	0.90

Data were presented by *n* (%) or median (interquartile range). *Glasgow Coma Scale score was available in 90 patients in non-stroke group.

**Table 3:** Multivariable analysis for time from last known well/symptom onset to imaging-based final diagnosis.

Variables	Exp (β)	95% CI	*p* value
Age (per 10-year increase)	0.95	0.87–1.03	0.20
Sex, male	0.86	0.68–1.08	0.19
Wake up symptom onset	3.34	2.61–4.28	<0.01
Initial activation of MET	0.71	0.56–0.91	<0.01
Symptoms first detected by physicians	1.37	0.96–1.95	0.08
Additional imaging required	1.26	0.99–1.59	0.06

Exp (β) indicates the rate of decrease in time from last known well/symptom onset to imaging-based final diagnosis. CI, confidence interval; MET, medical emergency team.

Overall, of the 183 patients, 77 (42.1%) had true strokes including 70 ischemic strokes, 5 intracerebral hemorrhages, and 2 subarachnoid hemorrhages. Potential causes of symptoms in patients without stroke were listed in Supplementary Table S3. In multivariable analysis, persistent neurological symptoms [odds ratio (OR) 1.44, 95% CI 1.24–1.68] and FAST-positive symptoms (OR 1.28, 95% CI 1.12–1.46) were associated with true stroke (Table 4). In analyses of individual presenting neurological symptom, motor symptoms (OR 2.61, 95% CI 1.35–5.04) and speech symptoms (OR 2.40, 95% CI 1.12–5.13) were associated with true stroke (Supplementary Figure 3).

**Table 4:** Multivariable analysis for true stroke.

Variables	OR	95% CI	*p* value
Age (per 10-year increase)	0.98	0.93–1.03	0.43
Sex, male	1.08	0.94–1.23	0.27
FAST-positive symptoms	1.28	1.12–1.46	<0.01
Persistent symptom	1.44	1.24–1.68	<0.01
History of stroke	0.90	0.78–1.04	0.14
Atrial fibrillation	1.17	0.99–1.39	0.07

CI, confidence interval; OR, odds ratio.

Among the 70 patients with ischemic stroke, 4 had transient symptoms. Among the 66 patients without transient symptoms, the median NIHSS score was 6 (IQR 3–15). Reperfusion therapy was performed in 17 patients (24.3%) including IVT only (*n* = 3), EVT only (*n* = 12), or both IVT and EVT (*n* = 2). Among the 61 patients who did not receive IVT, 56 patients (91.8%) had ≥1 contraindication for IVT. The frequency of contraindications and careful administrations for IVT was listed in Supplementary Table S4. The most common contraindication was exceeding the IVT time window (55.7%).

## Discussion

In this study, we found that wake-up symptom onset and initial MET activation were associated with diagnostic delay in patients with suspected in-hospital stroke. MRI was required in approximately three-quarters of patients to establish the final diagnosis. Persistent and FAST-positive symptoms were associated with true stroke.

Wake-up symptom onset was significantly associated with prolonged time to diagnosis. In this study, the estimated time from last known well to imaging-based final diagnosis was 810 min in the multivariable analysis mainly due to the longer time from last known well to symptom detection of 549 min. This finding is consistent with previous studies of out-of-hospital wake-up stroke which reported a median interval of approximately 7 h between last known well and symptom recognition and time to first imaging of mean 12.4 h (14, 15). Moreover, the overall time from last known well to detection of 120 min in this study was longer than that reported as 77.5 min in the previous study of in-hospital stroke (16), presumably because this study also included all patients with suspected in-hospital stroke in the non-stroke-specialized departments. Our findings may support the potential development of continuous neurological monitoring systems and AI-assisted stroke detection tools for hospitalized patients, particularly those at high risk of stroke (17, 18). An initial MET activation was associated with shorter diagnostic time. Early recognition of neurological symptoms as a medical emergency may facilitate prompt neuroimaging, neurological consultation, and timely diagnostic decision-making. Although our study was not designed to evaluate the effectiveness of MET activation itself, our findings support the potential role of an integrated in-hospital stroke response pathway in reducing diagnostic delay (7, 19, 20). As more than half of the patients in our cohort did not have stroke, neurological symptoms in hospitalized patients may reflect systemic conditions that mimic stroke or contribute to neurological deterioration. Therefore, an in-hospital code stroke system involving stroke specialists and MET, together with standardized neuroimaging, may facilitate timely stroke-specific management while maintaining systemic assessment and stabilization for patients with stroke mimics or other acute medical conditions. In addition, structured education and training for physicians and nurses outside stroke-specialized departments may improve recognition of stroke presentations and promote early activation of the stroke pathway. Most neurological symptoms were first detected by nurses. Our findings further support the efficacy of nurse-initiated in-hospital stroke response systems (21). Persistent and FAST-positive symptoms were associated with true stroke, which are consistent with previous studies in out-of-hospital stroke, where the FAST-screening tool remains one of the most reliable clinical indicators of acute stroke (22, 23). Our results suggest that these simple clinical features retain their diagnostic value in hospitalized patients and support the use of FAST-based education and triage protocols in non-stroke-specialized departments. Hospital-wide stroke awareness programs.

In this study, vast majority of true strokes were ischemic strokes, and a substantial proportion of patients required MRI following initial NCCT to establish the final diagnosis. The rate of intravenous thrombolysis remained relatively low in our cohort, consistent with previous reports of in-hospital stroke (1, 3, 4). Hospitalized patients frequently have competing medical conditions against IVT (24). However, an earlier diagnosis may facilitate appropriate patient selection and timely EVT for eligible patients. Previous studies have reported a higher frequency of stroke mimics among inpatients with suspected stroke than out-of-hospital stroke (25). Although our study was not designed to compare imaging strategies, MRI has superior sensitivity for detecting early ischemic lesions and may help differentiate stroke from mimics (26). An MRI-first approach may therefore be beneficial in selected hospitalized patients with suspected in-hospital stroke, particularly when MRI is rapidly available. In contrast, the use of CTA and CT perfusion was relatively limited in our cohort, which might have reflected an institutional workflow for contrast-enhanced imaging. Rapid vascular/perfusion imaging is important for identifying large-vessel occlusion and determining eligibility for EVT. With increasing evidence of EVT for patients with acute large-vessel occlusion stroke within 24 h onset, standardized in-hospital stroke pathways incorporating rapid CTA and CT perfusion may increase importance for hospitalized patients with suspected stroke. Unlike previous studies that focused on confirmed in-hospital stroke, our study included all patients in whom in-hospital stroke was clinically suspected. Therefore, our findings could be applicable to real-world in-hospital stroke triage. Improving recognition of neurological symptoms and establishing standardized in-hospital stroke pathways may reduce diagnostic delay and optimize acute stroke care in non-stroke-specialized departments.

This study has several limitations. First, this was a single-center retrospective study with missing variables, which may have introduced selection bias and limited the generalizability of the findings. Moreover, time metrics were calculated based on time stamps of electronic medical records, which might not reflect exact times of last known well or symptom detection. Second, patients were identified by screening head CT and MRI orders requested for the diagnosis or exclusion of suspected in-hospital stroke within 24 h of onset. Therefore, patients whose neurological symptoms were overlooked or not suggestive of stroke might not have been included. Third, this study was restricted to patients hospitalized outside stroke-specialized departments, and the findings may not be applicable to patients managed in stroke-specialized departments. Because neurological deterioration related to pre-existing neurological disorders may be difficult to distinguish from newly occurring stroke, further studies are warranted to evaluate diagnostic delays among patients with suspected in-hospital stroke in stroke-specialized departments. Fourth, the cause of neurological symptoms remained unclear in some patients without a final diagnosis of stroke. Finally, functional outcomes of patients with suspected in-hospital stroke were not assessed. Larger multicenter studies are warranted to confirm the generalizability of our findings and to investigate whether diagnostic delay affects treatment and functional outcomes in patients with suspected in-hospital stroke.

In conclusion, wake-up symptom onset and initial MET activation were associated with diagnostic delay, whereas persistent and FAST-positive symptoms were associated with true stroke. A standardized in-hospital stroke response pathway and education of non-stroke-specialized clinical staff may facilitate earlier recognition, appropriate triage, and timely diagnosis of suspected in-hospital stroke.

## Data Availability

The raw data supporting the conclusions of this article will be made available by the authors, without undue reservation.
