# Supplementary material for: Diagnostic delay and predictors of true stroke among patients with suspected in-hospital stroke: a single-center retrospective study
Source: Front Neurol. 2026 Oct 22;17:1979991. doi: 10.3389/fneur.2026.1979991 (PMC13735577; doi:10.3389/fneur.2026.1979991)
Supplement: Supplementary file 1 [file Data_Sheet_1.pdf]

Supplementary Material

Supplementary Table 1. Admitting departments

	N		N		N
Internal medicine	112	Surgery	49	Others	22
Cardiology	29	Orthopedic Surgery	13	Rehabilitation	16
General internal medicine	28	Cardiovascular Surgery	9	Psychiatry	6
Nephrology	14	Gastroenterological Surgery	5		
Gastroenterology	12	Ophthalmology	5		
Respiratory	11	Urology	5		
Hematology	8	Thoracic Surgery	4		
Rheumatology	7	Dermatology	4		
Endocrinology	3	Obstetrics and Gynecology	2		
		Otolaryngology	2		

Supplementary Table 2. Multivariable analysis for time from symptom detection to imaging-based final diagnosis

Variables	Exp (β)	95% CI	P value
Age (per 10-year increase)	0.95	0.86–1.05	0.33
Sex, male	0.86	0.65–1.13	0.28
Wake up symptom onset	1.44	1.07–1.94	0.02
Initial activation of MET	0.75	0.56–0.99	0.05
Symptoms first detected by other allied health professionals	1.76	1.02–3.05	0.04
Additional imaging required	1.72	1.30–2.28	<0.01

Exp (β) indicates the rate of decrease in time from last known well/symptom onset to imaging-based final diagnosis.

Abbreviations: CI, confidence interval; MET, medical emergency team.

Supplementary Table 3. Potential underlying cause of neurological symptoms in non-stroke patients

	N
Seizure	11
Peripheral neuropathy	9
Drug induced	9
Syncope/presyncope	8
Psychiatric disorder	4
Hyponatremia	4
Cachexia/brain metastasis	4
Hepatic encephalopathy	3
Delirium	2
Arthritis	2
Chronic subdural hematoma	2
Hypoglycemia	2
Infection/sepsis	2
Peripheral vertigo	2
Hypoxia/pulmonary embolism	2
Vitamin B1 deficiency	1
Unclear	39
Total	106

Supplementary Table 4. Contraindications and careful administrations for IVT

Contraindication	N
No diffusion-weighted imaging–fluid-attenuated inversion recovery mismatch mismatch within 4.5 h from symptom recognition, or not yet assessed	29
Stroke within 1 month	10
Major surgery or significant trauma within 14 days	9
Beyond 4.5 h from symptom onset or recognition	5
Concurrent hemorrhage	4
Infective endocarditis	3
Extensive early ischemic change	3
PT-INR >1.7	3
Concurrent acute aortic dissection	2
Prolonged aPTT	2
Non-traumatic intracranial hemorrhage	1
Within 4 h after the last dose of direct oral anticoagulant	1
Platelet ≤100,000/mm^3	1
**Careful administration**	**N**
Antithrombotic therapy	28
Age ≥81 years	21
Minor/rapidly improving symptoms	17
Stroke beyond 1 month of onset	11
Severe renal disorder (maintenance hemodialysis)	7
Thoracic aortic aneurysm	2
Trauma within 10 days	1
Convulsion	1

For 61 patients with ischemic stroke who did not undergo IVT.
Abbreviations: aPTT, activated partial thromboplastin time; IVT, intravenous thrombolysis; PT-INR, prothrombin time-international normalized ratio.

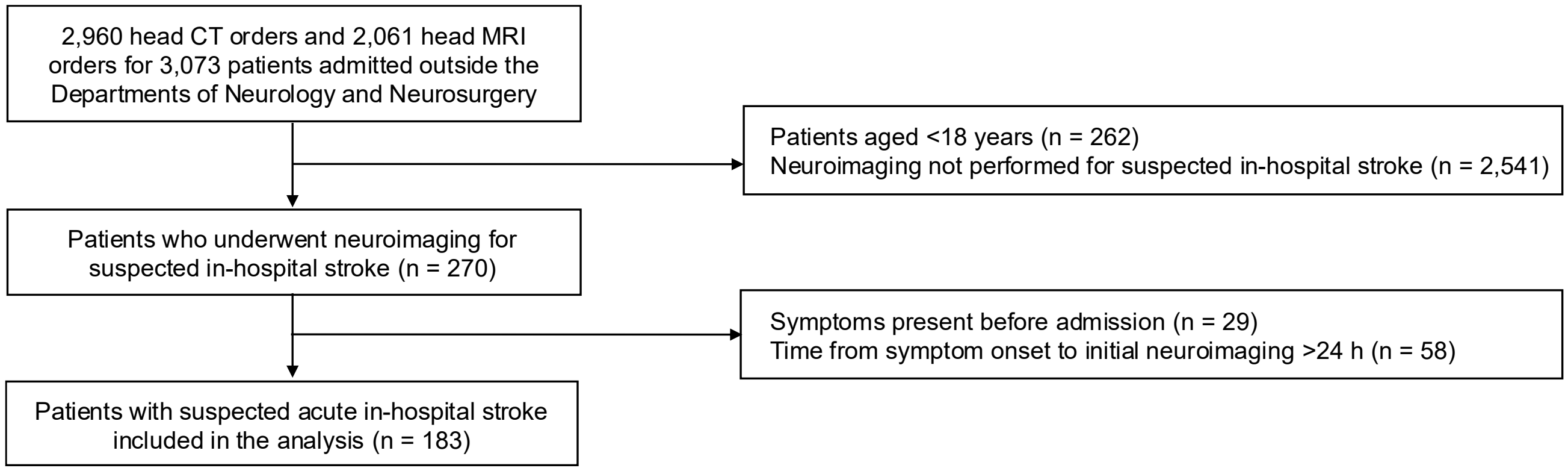

Supplementary Figure 1. Patient inclusion flowchart
Abbreviations: CT, computed tomography; MRI, magnetic resonance imaging.

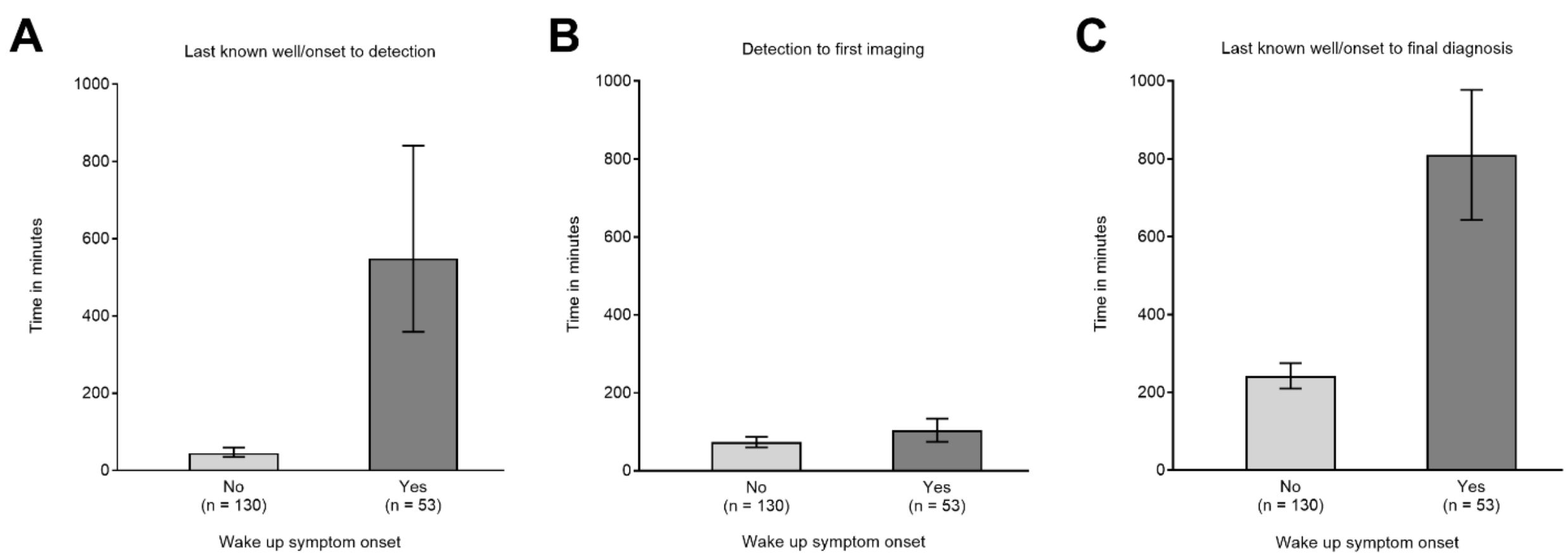

Supplementary Figure 2. Adjusted estimated time metrics in patients with suspected in-hospital stroke according to wake-up symptom onset.
Estimated time from (A) last known well/symptom onset to symptom detection, (B) symptom detection to first neuroimaging, and (C) last known well/symptom onset to imaging-based final diagnosis. Predicted values were adjusted for age, sex, and initial activation of the medical emergency team. Points represent the adjusted predicted mean, and error bars indicate the 95% confidence intervals.

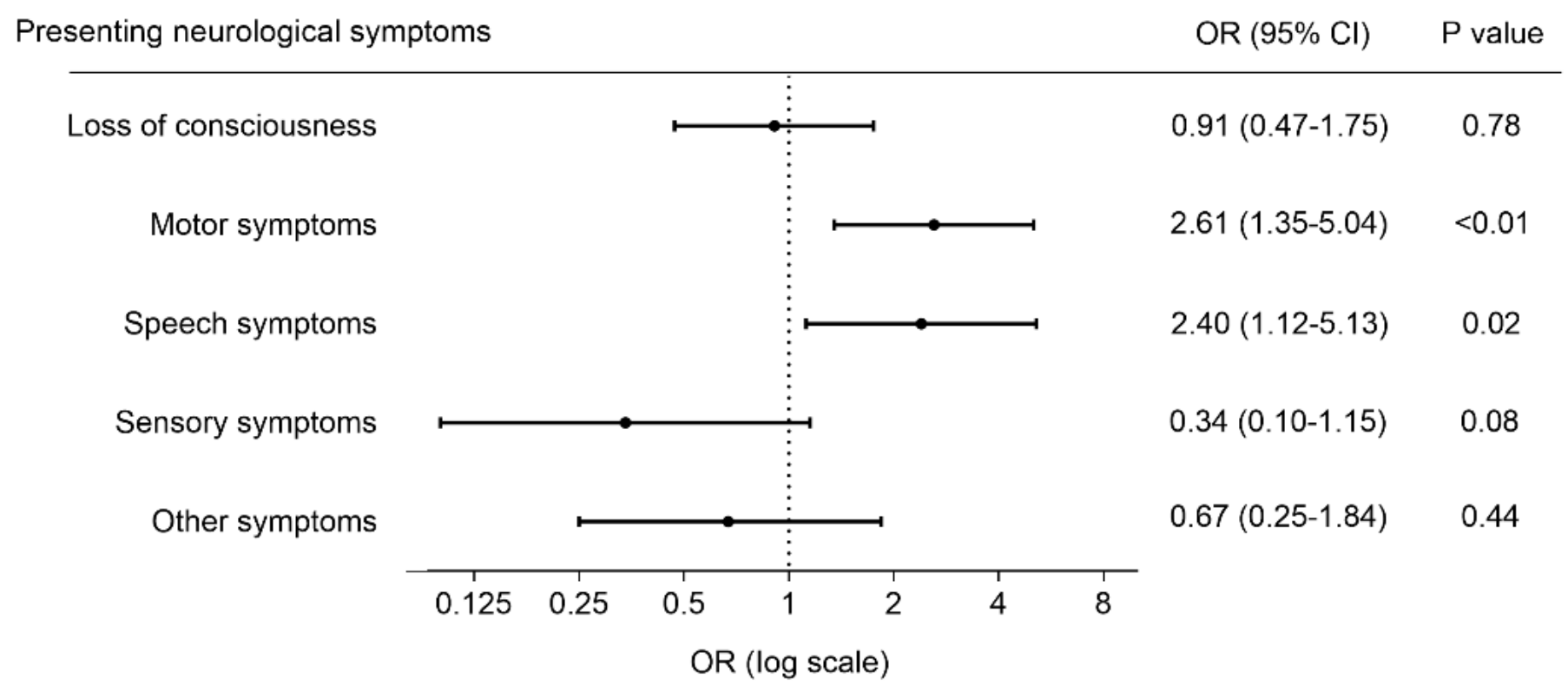

Supplementary Figure 3. Associations of presenting symptoms with true stroke among patients with suspected of in-hospital stroke

Models were adjusted with age, sex, and transient symptoms. ORs >1 indicate a higher likelihood of true stroke.

Abbreviations: CI, confidence intervals; OR, odds ratio.
